# *O*-Phenyl (cyclo­hexyl­amido)(*p*-tolyl­amido)­phosphinate

**DOI:** 10.1107/S1600536811018502

**Published:** 2011-05-25

**Authors:** Fahimeh Sabbaghi, Mehrdad Pourayoubi, Fatemeh Karimi Ahmadabad, Masood Parvez

**Affiliations:** a Department of Chemistry, Zanjan Branch, Islamic Azad University, PO Box 49195-467, Zanjan, Iran; b Department of Chemistry, Ferdowsi University of Mashhad, Mashhad, 91779, Iran; c Department of Chemistry, University of Calgary, 2500 University Drive, N.W., Calgary, Alberta, Canada T2N 1N4

## Abstract

In the title mol­ecule, C_19_H_25_N_2_O_2_P, the P atom is bonded in a distorted tetra­hedral environment. The dihedral angle between the two phenyl rings is 89.09 (8)°. The methyl H atoms are disordered over two sets of sites with equal occupancy. The O atom of the P=O group acts as a double hydrogen-bond acceptor of the type (N—H)_2_⋯(O=)P—, forming *R*_2_^2^(8) rings which are further linked into chains along [010].

## Related literature

For background to mixed-amido phosphinates, see: Pourayoubi *et al.* (2007 ▶). For double hydrogen-bond acceptors, see: Steiner (2002 ▶). For hydrogen-bond motifs, see: Bernstein *et al.* (1995 ▶).
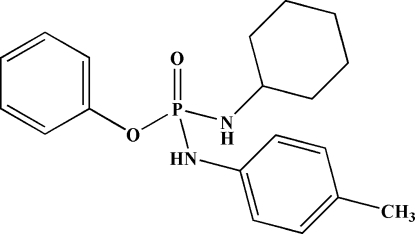


## Experimental

### 

#### Crystal data

C_19_H_25_N_2_O_2_P
*M* *_r_* = 344.38
Monoclinic, 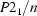

*a* = 15.5575 (4) Å
*b* = 7.7006 (3) Å
*c* = 16.1717 (4) Å
β = 108.9709 (17)°
*V* = 1832.17 (10) Å^3^
*Z* = 4
Mo *K*α radiation
μ = 0.16 mm^−1^
*T* = 173 K
0.16 × 0.14 × 0.10 mm

#### Data collection

Nonius KappaCCD diffractometer
Absorption correction: multi-scan (*SORTAV*; Blessing, 1997 ▶) *T*_min_ = 0.974, *T*_max_ = 0.984
13695 measured reflections
4172 independent reflections
3069 reflections with *I* > 2σ(*I*)
*R*_int_ = 0.070

#### Refinement

*R*[*F* ^2^ > 2σ(*F* ^2^)] = 0.056
*wR*(*F* ^2^) = 0.124
*S* = 1.06
4172 reflections
223 parameters
H atoms treated by a mixture of independent and constrained refinement
Δρ_max_ = 0.27 e Å^−3^
Δρ_min_ = −0.38 e Å^−3^

### 

Data collection: *COLLECT* (Hooft, 1998 ▶); cell refinement: *DENZO* (Otwinowski & Minor, 1997 ▶); data reduction: *SCALEPACK* (Otwinowski & Minor, 1997 ▶); program(s) used to solve structure: *SIR92* (Altomare *et al.*, 1993) ▶; program(s) used to refine structure: *SHELXL97* (Sheldrick, 2008 ▶); molecular graphics: *Mercury* (Macrae *et al.*, 2008 ▶); software used to prepare material for publication: *enCIFer* (Allen *et al.*, 2004 ▶).

## Supplementary Material

Crystal structure: contains datablocks I, global. DOI: 10.1107/S1600536811018502/lh5250sup1.cif

Structure factors: contains datablocks I. DOI: 10.1107/S1600536811018502/lh5250Isup2.hkl

Supplementary material file. DOI: 10.1107/S1600536811018502/lh5250Isup3.cml

Additional supplementary materials:  crystallographic information; 3D view; checkCIF report

## Figures and Tables

**Table 1:** Hydrogen-bond geometry (Å, °)

*D*—H⋯*A*	*D*—H	H⋯*A*	*D*⋯*A*	*D*—H⋯*A*
N1—H1*N*⋯O1^i^	0.81 (3)	2.16 (3)	2.961 (3)	169 (3)
N2—H2*N*⋯O1^ii^	0.84 (3)	2.20 (3)	3.023 (3)	167 (2)

Symmetry codes: (i) 
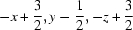
; (ii) 
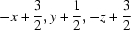
.

**Fig. 1.**
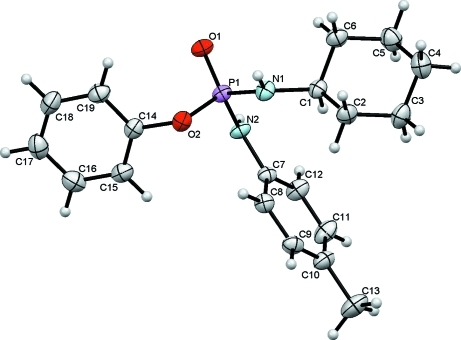
The molecular structure of the title compound with ellipsoids shown at the 50% probability level. The disorder is not shown.

**Fig. 2.**
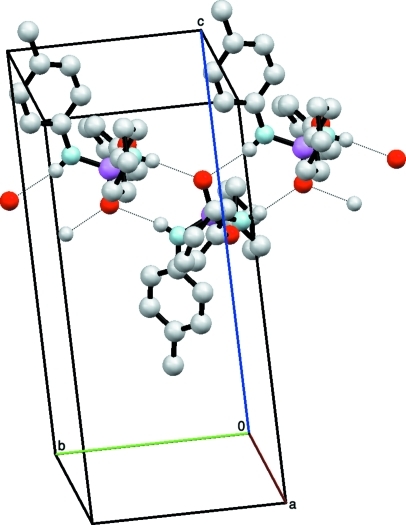
Part of the crystal structure of the title compound with hydrogen bonding shown as dotted lines (the C—H hydrogen atoms are omitted for clarity).

## supplementary crystallographic information

### Comment

The structure determination of title compound was performed as a part of a project in our laboratory on the synthesis of new mixed-amido phosphinates having a P(O)(O)(N')(N") skeleton (Pourayoubi *et al.*, 2007).

The P═O, P—O and P—N bond lengths are standard for this type of compound (Pourayoubi *et al.*, 2007). The P atom has a distorted tetrahedral configuration (Fig. 1) with the bond angles in the range of 98.55 (10)° [O2–P1–N1] to 119.80 (10)° [O1–P1–N1].

The phosphoryl group respectively adopts a *syn* orientation with respect to the N–H unit of *p*-tolylamido moiety and a *gauche* position relative to that of cyclohexylamido substituent.

In the crystal structure, the molecules are linked by two intermolecular N—H···OP hydrogen bonds (Table 1) into chains in the direction of the *b* axis in which the O atom of the P═O group acts as a double H-acceptor (Steiner, 2002) (Fig. 2). From this arrangement *R*_2_^2^(8) rings are formed (Bernstein *et al.*, 1995).

### Experimental

To a solution of (C_6_H_5_O)(4-CH_3_C_6_H_4_NH)P(O)Cl in chloroform, a solution of cyclohexylamine (1:2 mole ratio) in chloroform was added at 273 K. After 4 h stirring, the solvent was removed and product was washed with distilled water. Single crystals were obtained from a solution of the title compound in CH_3_CN/CHCl_3_ after slow evaporation at room temperature.

### Refinement

Though the H-atoms were visible in difference fourier maps they were included in geometrically idealized positions with C—H distances = 0.95, 0.98, 0.99 and 1.00 Å for aryl, methyl, methylene and methine type H-atoms, respectively. The methyl H-atoms are disordered over six sites with equal site occupancy factors. The H-atoms bonded to N-atoms were allowed to refine. The H-atoms were assigned *U*_iso_ = 1.5 times *U*_eq_ methyl C atom and 1.2 times *U*_eq_ of the rest of the parent atoms (C/N).

### Crystal data

**Table:**

C_19_H_25_N_2_O_2_P	*F*(000) = 736
*M**_r_* = 344.38	*D*_x_ = 1.248 Mg m^−^^3^
Monoclinic, *P*2_1_/*n*	Mo *K*α radiation, λ = 0.71073 Å
Hall symbol: -P 2yn	Cell parameters from 7090 reflections
*a* = 15.5575 (4) Å	θ = 3.0–27.5°
*b* = 7.7006 (3) Å	µ = 0.16 mm^−^^1^
*c* = 16.1717 (4) Å	*T* = 173 K
β = 108.9709 (17)°	Prism, colorless
*V* = 1832.17 (10) Å^3^	0.16 × 0.14 × 0.10 mm
*Z* = 4	

### Data collection

**Table:**

Nonius KappaCCD diffractometer	4172 independent reflections
Radiation source: fine-focus sealed tube	3069 reflections with *I* > 2σ(*I*)
graphite	*R*_int_ = 0.070
ω and φ scans	θ_max_ = 27.6°, θ_min_ = 3.0°
Absorption correction: multi-scan (*SORTAV*; Blessing, 1997)	*h* = −20→20
*T*_min_ = 0.974, *T*_max_ = 0.984	*k* = −9→9
13695 measured reflections	*l* = −20→21

### Refinement

**Table:**

Refinement on *F*^2^	Primary atom site location: structure-invariant direct methods
Least-squares matrix: full	Secondary atom site location: difference Fourier map
*R*[*F*^2^ > 2σ(*F*^2^)] = 0.056	Hydrogen site location: inferred from neighbouring sites
*wR*(*F*^2^) = 0.124	H atoms treated by a mixture of independent and constrained refinement
*S* = 1.06	*w* = 1/[σ^2^(*F*_o_^2^) + (0.0234*P*)^2^ + 2.302*P*] where *P* = (*F*_o_^2^ + 2*F*_c_^2^)/3
4172 reflections	(Δ/σ)_max_ < 0.001
223 parameters	Δρ_max_ = 0.27 e Å^−^^3^
0 restraints	Δρ_min_ = −0.38 e Å^−^^3^

### Special details

**Table:**

Geometry. All e.s.d.'s (except the e.s.d. in the dihedral angle between two l.s. planes) are estimated using the full covariance matrix. The cell e.s.d.'s are taken into account individually in the estimation of e.s.d.'s in distances, angles and torsion angles; correlations between e.s.d.'s in cell parameters are only used when they are defined by crystal symmetry. An approximate (isotropic) treatment of cell e.s.d.'s is used for estimating e.s.d.'s involving l.s. planes.
Refinement. Refinement of *F*^2^ against ALL reflections. The weighted *R*-factor *wR* and goodness of fit *S* are based on *F*^2^, conventional *R*-factors *R* are based on *F*, with *F* set to zero for negative *F*^2^. The threshold expression of *F*^2^ > σ(*F*^2^) is used only for calculating *R*-factors(gt) *etc*. and is not relevant to the choice of reflections for refinement. *R*-factors based on *F*^2^ are statistically about twice as large as those based on *F*, and *R*- factors based on ALL data will be even larger.

### Fractional atomic coordinates and isotropic or equivalent isotropic displacement parameters (Å^2^)

**Table:**

	*x*	*y*	*z*	*U*_iso_*/*U*_eq_	Occ. (<1)
P1	0.73253 (4)	0.16106 (8)	0.67651 (4)	0.02345 (15)	
O1	0.71919 (11)	0.1863 (2)	0.76217 (10)	0.0283 (4)	
O2	0.64631 (10)	0.0713 (2)	0.60756 (10)	0.0285 (4)	
N1	0.80997 (14)	0.0288 (3)	0.66811 (13)	0.0273 (4)	
H1N	0.8023 (17)	−0.070 (4)	0.6810 (17)	0.033*	
N2	0.75059 (13)	0.3485 (3)	0.63724 (12)	0.0247 (4)	
H2N	0.7553 (17)	0.434 (3)	0.6707 (16)	0.030*	
C1	0.90574 (15)	0.0755 (3)	0.68466 (15)	0.0261 (5)	
H1	0.9074	0.1999	0.6674	0.031*	
C2	0.94454 (16)	−0.0325 (4)	0.62658 (16)	0.0335 (6)	
H2A	0.9434	−0.1568	0.6419	0.040*	
H2B	0.9063	−0.0178	0.5647	0.040*	
C3	1.04166 (17)	0.0216 (4)	0.63741 (18)	0.0402 (6)	
H3A	1.0666	−0.0555	0.6018	0.048*	
H3B	1.0418	0.1418	0.6158	0.048*	
C4	1.10170 (18)	0.0119 (4)	0.73256 (18)	0.0432 (7)	
H4A	1.1089	−0.1110	0.7515	0.052*	
H4B	1.1627	0.0584	0.7382	0.052*	
C5	1.06180 (17)	0.1142 (4)	0.79143 (17)	0.0410 (7)	
H5A	1.0629	0.2395	0.7781	0.049*	
H5B	1.0998	0.0965	0.8531	0.049*	
C6	0.96421 (16)	0.0595 (4)	0.77984 (15)	0.0329 (6)	
H6A	0.9635	−0.0622	0.7994	0.040*	
H6B	0.9391	0.1340	0.8164	0.040*	
C7	0.77827 (15)	0.3772 (3)	0.56278 (14)	0.0236 (5)	
C8	0.75810 (16)	0.2609 (3)	0.49355 (15)	0.0275 (5)	
H8	0.7229	0.1600	0.4936	0.033*	
C9	0.79007 (16)	0.2939 (3)	0.42412 (15)	0.0299 (5)	
H9	0.7767	0.2132	0.3772	0.036*	
C10	0.84044 (17)	0.4393 (3)	0.42080 (16)	0.0333 (6)	
C11	0.85877 (19)	0.5555 (4)	0.49027 (18)	0.0396 (6)	
H11	0.8929	0.6574	0.4896	0.048*	
C12	0.82810 (17)	0.5252 (3)	0.56052 (16)	0.0320 (6)	
H12	0.8413	0.6063	0.6073	0.038*	
C13	0.8741 (2)	0.4732 (4)	0.34463 (18)	0.0489 (8)	
H13A	0.9083	0.5823	0.3543	0.073*	0.50
H13B	0.8221	0.4817	0.2907	0.073*	0.50
H13C	0.9136	0.3776	0.3395	0.073*	0.50
H13D	0.8544	0.3788	0.3020	0.073*	0.50
H13E	0.9406	0.4794	0.3656	0.073*	0.50
H13F	0.8491	0.5835	0.3168	0.073*	0.50
C14	0.55737 (15)	0.1271 (3)	0.59596 (15)	0.0264 (5)	
C15	0.51721 (17)	0.2434 (3)	0.52978 (15)	0.0328 (6)	
H15	0.5507	0.2917	0.4956	0.039*	
C16	0.42679 (18)	0.2884 (4)	0.51410 (17)	0.0401 (6)	
H16	0.3980	0.3682	0.4687	0.048*	
C17	0.37857 (18)	0.2181 (4)	0.56396 (18)	0.0412 (7)	
H17	0.3169	0.2497	0.5532	0.049*	
C18	0.42027 (17)	0.1015 (4)	0.62967 (17)	0.0390 (6)	
H18	0.3866	0.0522	0.6635	0.047*	
C19	0.51073 (17)	0.0554 (3)	0.64696 (16)	0.0336 (6)	
H19	0.5397	−0.0235	0.6927	0.040*	

### Atomic displacement parameters (Å^2^)

**Table:**

	*U*^11^	*U*^22^	*U*^33^	*U*^12^	*U*^13^	*U*^23^
P1	0.0297 (3)	0.0183 (3)	0.0243 (3)	−0.0017 (2)	0.0115 (2)	−0.0006 (2)
O1	0.0396 (9)	0.0227 (9)	0.0261 (8)	−0.0002 (7)	0.0155 (7)	0.0006 (7)
O2	0.0299 (8)	0.0265 (9)	0.0315 (9)	−0.0031 (7)	0.0132 (7)	−0.0050 (7)
N1	0.0316 (10)	0.0179 (10)	0.0344 (11)	−0.0028 (9)	0.0136 (9)	0.0009 (9)
N2	0.0356 (11)	0.0199 (10)	0.0226 (10)	−0.0011 (9)	0.0149 (8)	−0.0012 (8)
C1	0.0284 (11)	0.0216 (12)	0.0303 (12)	−0.0008 (10)	0.0122 (10)	0.0010 (10)
C2	0.0347 (13)	0.0365 (15)	0.0310 (13)	0.0007 (11)	0.0128 (11)	−0.0065 (11)
C3	0.0386 (14)	0.0436 (17)	0.0460 (16)	0.0003 (13)	0.0242 (13)	−0.0058 (13)
C4	0.0310 (13)	0.0441 (17)	0.0534 (17)	0.0016 (12)	0.0124 (13)	−0.0047 (14)
C5	0.0353 (14)	0.0447 (17)	0.0396 (15)	−0.0025 (13)	0.0074 (12)	−0.0071 (13)
C6	0.0382 (14)	0.0329 (14)	0.0297 (12)	0.0000 (11)	0.0138 (11)	−0.0028 (11)
C7	0.0270 (11)	0.0205 (12)	0.0235 (11)	0.0027 (9)	0.0085 (9)	0.0024 (9)
C8	0.0295 (12)	0.0238 (12)	0.0292 (12)	−0.0008 (10)	0.0095 (10)	−0.0009 (10)
C9	0.0355 (13)	0.0304 (14)	0.0240 (11)	0.0017 (11)	0.0098 (10)	−0.0043 (10)
C10	0.0402 (14)	0.0335 (14)	0.0322 (13)	0.0040 (12)	0.0201 (11)	0.0041 (11)
C11	0.0514 (16)	0.0282 (15)	0.0486 (16)	−0.0080 (12)	0.0290 (13)	−0.0009 (12)
C12	0.0444 (14)	0.0220 (12)	0.0342 (13)	−0.0069 (11)	0.0190 (11)	−0.0049 (10)
C13	0.0657 (19)	0.0486 (19)	0.0457 (16)	−0.0036 (16)	0.0366 (15)	−0.0001 (14)
C14	0.0300 (12)	0.0219 (12)	0.0292 (12)	−0.0046 (10)	0.0122 (10)	−0.0062 (10)
C15	0.0398 (14)	0.0319 (14)	0.0290 (12)	−0.0039 (11)	0.0142 (11)	−0.0003 (11)
C16	0.0409 (15)	0.0403 (16)	0.0347 (14)	0.0051 (13)	0.0063 (12)	0.0006 (12)
C17	0.0308 (13)	0.0462 (17)	0.0460 (16)	0.0017 (12)	0.0115 (12)	−0.0098 (14)
C18	0.0383 (14)	0.0424 (17)	0.0433 (15)	−0.0078 (13)	0.0229 (12)	−0.0044 (13)
C19	0.0401 (14)	0.0291 (14)	0.0334 (13)	−0.0031 (11)	0.0146 (11)	0.0011 (11)

### Geometric parameters (Å, °)

**Table:**

P1—O1	1.4788 (16)	C7—C8	1.388 (3)
P1—O2	1.5961 (17)	C8—C9	1.391 (3)
P1—N1	1.617 (2)	C8—H8	0.9500
P1—N2	1.637 (2)	C9—C10	1.378 (4)
O2—C14	1.402 (3)	C9—H9	0.9500
N1—C1	1.470 (3)	C10—C11	1.391 (4)
N1—H1N	0.81 (3)	C10—C13	1.510 (3)
N2—C7	1.422 (3)	C11—C12	1.387 (3)
N2—H2N	0.84 (3)	C11—H11	0.9500
C1—C6	1.517 (3)	C12—H12	0.9500
C1—C2	1.519 (3)	C13—H13A	0.9800
C1—H1	1.0000	C13—H13B	0.9800
C2—C3	1.522 (3)	C13—H13C	0.9800
C2—H2A	0.9900	C13—H13D	0.9800
C2—H2B	0.9900	C13—H13E	0.9800
C3—C4	1.520 (4)	C13—H13F	0.9800
C3—H3A	0.9900	C14—C15	1.379 (3)
C3—H3B	0.9900	C14—C19	1.379 (3)
C4—C5	1.515 (4)	C15—C16	1.390 (4)
C4—H4A	0.9900	C15—H15	0.9500
C4—H4B	0.9900	C16—C17	1.378 (4)
C5—C6	1.527 (3)	C16—H16	0.9500
C5—H5A	0.9900	C17—C18	1.381 (4)
C5—H5B	0.9900	C17—H17	0.9500
C6—H6A	0.9900	C18—C19	1.389 (4)
C6—H6B	0.9900	C18—H18	0.9500
C7—C12	1.386 (3)	C19—H19	0.9500
			
O1—P1—O2	111.68 (9)	C7—C8—H8	120.4
O1—P1—N1	119.80 (10)	C9—C8—H8	120.4
O2—P1—N1	98.55 (10)	C10—C9—C8	122.5 (2)
O1—P1—N2	109.97 (10)	C10—C9—H9	118.8
O2—P1—N2	108.77 (10)	C8—C9—H9	118.8
N1—P1—N2	107.23 (10)	C9—C10—C11	117.5 (2)
C14—O2—P1	121.97 (14)	C9—C10—C13	121.7 (2)
C1—N1—P1	124.70 (17)	C11—C10—C13	120.9 (2)
C1—N1—H1N	114.1 (19)	C12—C11—C10	121.2 (2)
P1—N1—H1N	113.3 (19)	C12—C11—H11	119.4
C7—N2—P1	127.10 (16)	C10—C11—H11	119.4
C7—N2—H2N	115.9 (18)	C11—C12—C7	120.3 (2)
P1—N2—H2N	115.5 (17)	C11—C12—H12	119.9
N1—C1—C6	113.73 (19)	C7—C12—H12	119.9
N1—C1—C2	109.64 (19)	C10—C13—H13A	109.5
C6—C1—C2	110.8 (2)	C10—C13—H13B	109.5
N1—C1—H1	107.5	H13A—C13—H13B	109.5
C6—C1—H1	107.5	C10—C13—H13C	109.5
C2—C1—H1	107.5	H13A—C13—H13C	109.5
C3—C2—C1	111.0 (2)	H13B—C13—H13C	109.5
C3—C2—H2A	109.4	C10—C13—H13D	109.5
C1—C2—H2A	109.4	H13A—C13—H13D	141.1
C3—C2—H2B	109.4	H13B—C13—H13D	56.3
C1—C2—H2B	109.4	H13C—C13—H13D	56.3
H2A—C2—H2B	108.0	C10—C13—H13E	109.5
C4—C3—C2	111.3 (2)	H13A—C13—H13E	56.3
C4—C3—H3A	109.4	H13B—C13—H13E	141.1
C2—C3—H3A	109.4	H13C—C13—H13E	56.3
C4—C3—H3B	109.4	H13D—C13—H13E	109.5
C2—C3—H3B	109.4	C10—C13—H13F	109.5
H3A—C3—H3B	108.0	H13A—C13—H13F	56.3
C5—C4—C3	111.6 (2)	H13B—C13—H13F	56.3
C5—C4—H4A	109.3	H13C—C13—H13F	141.1
C3—C4—H4A	109.3	H13D—C13—H13F	109.5
C5—C4—H4B	109.3	H13E—C13—H13F	109.5
C3—C4—H4B	109.3	C15—C14—C19	122.0 (2)
H4A—C4—H4B	108.0	C15—C14—O2	118.8 (2)
C4—C5—C6	111.9 (2)	C19—C14—O2	119.0 (2)
C4—C5—H5A	109.2	C14—C15—C16	118.7 (2)
C6—C5—H5A	109.2	C14—C15—H15	120.7
C4—C5—H5B	109.2	C16—C15—H15	120.7
C6—C5—H5B	109.2	C17—C16—C15	120.4 (3)
H5A—C5—H5B	107.9	C17—C16—H16	119.8
C1—C6—C5	110.2 (2)	C15—C16—H16	119.8
C1—C6—H6A	109.6	C16—C17—C18	119.8 (2)
C5—C6—H6A	109.6	C16—C17—H17	120.1
C1—C6—H6B	109.6	C18—C17—H17	120.1
C5—C6—H6B	109.6	C17—C18—C19	120.9 (2)
H6A—C6—H6B	108.1	C17—C18—H18	119.6
C12—C7—C8	119.4 (2)	C19—C18—H18	119.6
C12—C7—N2	118.5 (2)	C14—C19—C18	118.2 (2)
C8—C7—N2	122.1 (2)	C14—C19—H19	120.9
C7—C8—C9	119.1 (2)	C18—C19—H19	120.9
			
O1—P1—O2—C14	46.41 (19)	C12—C7—C8—C9	1.3 (3)
N1—P1—O2—C14	173.31 (17)	N2—C7—C8—C9	−177.7 (2)
N2—P1—O2—C14	−75.14 (18)	C7—C8—C9—C10	−0.7 (4)
O1—P1—N1—C1	−86.5 (2)	C8—C9—C10—C11	−0.2 (4)
O2—P1—N1—C1	152.36 (18)	C8—C9—C10—C13	−179.7 (2)
N2—P1—N1—C1	39.6 (2)	C9—C10—C11—C12	0.5 (4)
O1—P1—N2—C7	170.92 (18)	C13—C10—C11—C12	−180.0 (3)
O2—P1—N2—C7	−66.5 (2)	C10—C11—C12—C7	0.1 (4)
N1—P1—N2—C7	39.1 (2)	C8—C7—C12—C11	−1.0 (4)
P1—N1—C1—C6	86.5 (2)	N2—C7—C12—C11	178.0 (2)
P1—N1—C1—C2	−148.82 (18)	P1—O2—C14—C15	95.6 (2)
N1—C1—C2—C3	176.0 (2)	P1—O2—C14—C19	−88.8 (2)
C6—C1—C2—C3	−57.7 (3)	C19—C14—C15—C16	−0.3 (4)
C1—C2—C3—C4	55.5 (3)	O2—C14—C15—C16	175.2 (2)
C2—C3—C4—C5	−53.6 (3)	C14—C15—C16—C17	0.1 (4)
C3—C4—C5—C6	54.0 (3)	C15—C16—C17—C18	−0.3 (4)
N1—C1—C6—C5	−178.7 (2)	C16—C17—C18—C19	0.7 (4)
C2—C1—C6—C5	57.2 (3)	C15—C14—C19—C18	0.7 (4)
C4—C5—C6—C1	−55.6 (3)	O2—C14—C19—C18	−174.8 (2)
P1—N2—C7—C12	−151.43 (19)	C17—C18—C19—C14	−0.9 (4)
P1—N2—C7—C8	27.6 (3)		

### Hydrogen-bond geometry (Å, °)

**Table:**

*D*—H···*A*	*D*—H	H···*A*	*D*···*A*	*D*—H···*A*
N1—H1N···O1^i^	0.81 (3)	2.16 (3)	2.961 (3)	169 (3)
N2—H2N···O1^ii^	0.84 (3)	2.20 (3)	3.023 (3)	167 (2)

Symmetry codes: (i) −*x*+3/2, *y*−1/2, −*z*+3/2; (ii) −*x*+3/2, *y*+1/2, −*z*+3/2.
